# Usefulness of two-dimensional shear wave elastography in the assessment of non-alcoholic fatty liver disease in children and adolescents

**DOI:** 10.1038/s41598-023-37281-z

**Published:** 2023-06-21

**Authors:** Jong Seo Yoon, Kyoung Ja Lim, Il Tae Hwang

**Affiliations:** 1 Department of Paediatrics, Hallym University Kangdong Sacred Heart Hospital, Hallym University College of Medicine, Seoul, South Korea; 2 Department of Radiology, Hallym University Kangdong Sacred Heart Hospital, Hallym University College of Medicine, Seoul, 05355 South Korea

**Keywords:** Liver diseases, Paediatric research, Non-alcoholic fatty liver disease

## Abstract

Two-dimensional shear wave elastography (2D-SWE) evaluates liver stiffness using a non-invasive method, but studies in the paediatric population are rare. This study evaluated the role of 2D-SWE in the diagnosis and severity of paediatric non-alcoholic fatty liver disease (NAFLD). In total, 131 patients with NAFLD and 25 healthy controls were enrolled in this study. The diagnosis and severity of NAFLD were initially assessed using the ultrasound fatty liver index (US-FLI), and all participants underwent 2D-SWE. US-FLI semi-quantitatively measures the severity of NAFLD on a scale of 2–8. The assessment of liver stiffness measurement (LSM) by 2D-SWE is presented in kilopascals (kPa). The NAFLD group was characterised by significantly higher LSM (4.40 ± 0.90 kPa) than the control group (3.76 ± 0.28 kPa) (*P* < 0.001). 2D-SWE significantly correlated with age, height, weight, body mass index, glucose, aspartate aminotransferase, alanine aminotransferase, high-density lipoprotein cholesterol, US-FLI, and triglyceride-glucose index (*P* < 0.001). In the receiver operating characteristic curve analysis, the area under the curve of LSM for predicting US-FLI ≥ 2 and ≥ 6 was 0.784 (*P* < 0.001) and 0.819 (*P* < 0.001), respectively. In conclusion, we suggest that 2D-SWE can be used as a non-invasive diagnostic tool for diagnosing and assessing the severity of paediatric NAFLD.

## Introduction

Non-alcoholic fatty liver disease (NAFLD) is defined as chronic hepatic steatosis in the absence of any other cause of secondary fat accumulation in the liver, including alcohol consumption, viral infection, or autoimmune disease^1^. Obesity is a risk factor for NAFLD, and the incidence of NAFLD in children is increasing worldwide, as in adults, which parallels the rise in obesity prevalence^2–5^. The recent COVID-19 pandemic has raised concerns about worsening obesity prevalence^6–8^. NAFLD that begins and progresses in childhood may be more severe than that in adults. Therefore, early detection and prevention of paediatric NAFLD progression are important^9,10^. Traditionally, the gold standard for the diagnosis and staging of NAFLD has been liver biopsy, but it has limitations due to its invasive nature and associated complications. Therefore, researchers are interested in alternative tests that can represent liver biopsy results^11^.

As a non-invasive imaging alternative, ultrasound (US) is widely utilized in routine NAFLD examinations. New US methods for screening and monitoring the NAFLD spectrum are being introduced. Recently, studies have been conducted to predict the grades of steatosis, non-alcoholic steatohepatitis (NASH), and fibrosis in clinical practice using semi-quantitative US scoring systems and US elastography. Non-invasive imaging alternatives are necessary screening methods for paediatric NAFLD, but the usefulness of these new US methods has been reported primarily in the adult population, and few studies have been conducted in paediatric populations, increasing the need for further studies. Ultrasound fatty liver indicator (US-FLI) is a semi-quantitative method that represents the status of NAFLD using a scoring system. Classically, US-based steatosis evaluation had limitations by presenting subjective diagnostic findings such as mild, moderate, or severe, but US-FLI has the advantage of being able to present objective diagnostic findings with NAFLD scores between 2 and 8. The US-FLI score showed good discriminating power for various grades of steatosis, and a score of less than 4 showed a high negative predictive value that could exclude the diagnosis of severe NASH, thereby avoiding unnecessary liver biopsy in adults^12^. Recently, it was reported that US-FLI can predict hepatitis in children with NAFLD, providing a basis for applying US-FLI to paediatric NAFLD^13^. Another ultrasound technique, ultrasonography-based elastography techniques evaluate liver stiffness measurement (LSM) to assess liver fibrosis in NAFLD. 2D-SWE is a recently developed non-invasive method to evaluate liver stiffness and was first introduced in 2005. 2D-SWE measures the propagation velocity of acoustically generated shear waves in tissue and evaluates liver stiffness in real time, as shear waves are generated by US pulses with simultaneous anatomical B-mode US imaging. Shear wave velocity measurements yield qualitative and quantitative estimates of tissue elasticity. And the quantitative elasticity image is displayed as a 2D colour map, where each colour codes the elasticity (kPa) as a quantitative result. 2D-SWE showed good diagnostic performance for advanced fibrosis, but studies on 2D-SWE related to NAFLD in the paediatric population are limited^14^.

Our research team considered 2D-SWE as a useful, non-invasive tool to sequentially follow the progression of NAFLD, which begins in childhood and progresses to fibrosis. Although existing research results have limitations in accuracy differences due to population differences and potential confounding variables when applied to this study group, we hypothesized that US-FLI and 2D-SWE were sufficient to diagnose the presence of NAFLD and further correlate it with the severity of NAFLD. Moreover, since the severity of NAFLD increases as the TyG index, a surrogate marker of insulin resistance, increases^15^, we hypothesised that if 2D-SWE reflects the severity of NAFLD, it will also be positively associated with insulin resistance surrogate markers. Therefore, this study investigated the usefulness of 2D-SWE for the detection and assessment of disease severity in children with NAFLD and the association between 2D-SWE and insulin resistance in NAFLD.

## Results

### Clinical characteristics of controls and patients with NAFLD

The clinical characteristics of the controls and patients with NAFLD are presented as mean ± standard deviation (Table 1). Among the 156 participants, 25 were controls and 131 were patients with NAFLD. The mean ages of the control and NAFLD groups were 8.12 ± 1.22 and 11.57 ± 2.29 years, respectively. Age, height, height standard deviation score (SDS), weight, weight SDS, body mass index (BMI), and BMI SDS were significantly higher in the NAFLD group (*P* < 0.001). The levels of aspartate aminotransferase (AST), alanine aminotransferase (ALT), low-density lipoprotein cholesterol (LDL-C), non-HDL-C, triglyceride (TG), insulin, TyG index, US-FLI, and liver stiffness measurement (LSM) were significantly elevated in the NAFLD group, whereas high-density lipoprotein cholesterol (HDL-C) levels were significantly decreased (*P* < 0.001). The glucose and total cholesterol levels did not differ between the two groups.

**Table 1:** Clinical characteristics of controls and patients with NAFLD.

	Controls (n = 25)	NAFLD (n = 131)	*P*
Age, years	8.12 ± 1.22	11.57 ± 2.29	< 0.001
Height, cm	125.20 ± 7.66	152.24 ± 7.66	< 0.001
Height SDS	− 0.45 ± 0.800	0.87 ± 1.20	< 0.001
Weight, kg	26.82 ± 4.70	63.78 ± 19.01	< 0.001
Weight SDS	− 0.14 ± 0.57	2.23 ± 1.12	< 0.001
BMI, kg/m^2^	16.99 ± 1.42	26.91 ± 4.31	< 0.001
BMI SDS	0.10 ± 0.59	2.45 ± 1.02	< 0.001
Glucose, mg/dL	94.56 ± 7.33	100.74 ± 25.25	0.228
AST, IU/L	31.04 ± 6.17	53.44 ± 30.91	< 0.001
ALT, IU/L	16.52 ± 5.99	86.13 ± 56.19	< 0.001
TC, mg/dL	172.44 ± 27.55	179.43 ± 58.27	0.559
LDL-C, mg/dL	95.40 ± 16.03	109.12 ± 24.43	0.001
HDL-C, mg/dL	65.12 ± 10.60	48.39 ± 11.83	< 0.001
Triglycerides, mg/dL	64.76 ± 18.48	131.04 ± 74.74	< 0.001
TyG index	7.98 ± 0.32	8.65 ± 0.57	< 0.001
US-FLI	0	3.96 ± 1.29	< 0.001
LSM, kPa	3.76 ± 0.28	4.40 ± 0.90	< 0.001

*BMI* body mass index, *SDS* standard deviation scores, *AST* aspartate transaminase, *ALT* alanine aminotransferase, *TC* total cholesterol, *LDL-C* low-density lipoprotein cholesterol, *HDL-C* high-density lipoprotein cholesterol, *TyG index* triglyceride glucose index, *US-FLI* ultrasonographic fatty liver indicator, *LSM* liver stiffness measurement.

### The correlation between LSM and clinical parameters

Table 2 shows the results of Pearson’s correlation analysis. Age (r = 0.415, *P* < 0.001), height (r = 0.409, *P* < 0.001), height SDS (r = 0.224, *P* = 0.005), weight (r = 0.521, *P* < 0.001), weight SDS (r = 0.458, *P* < 0.001), BMI (r = 0.529, *P* < 0.001), BMI-SDS (r = 0.481, *P* < 0.001), glucose (r = 0.351, *P* < 0.001), AST (r = 0.364, *P* < 0.001), ALT (r = 0.291, *P* < 0.001), TG (r = 0.221, *P* = 0.009), TyG index (r = 0.313, *P* < 0.001), and US-FLI (r = 0.422, *P* < 0.001) were positively correlated with LSM, whereas HDL-C (r = − 0.334, *P* < 0.001) showed a negative correlation with LSM. Sex, TC, and LDL-C did not correlate with LSM.

**Table 2:** The correlation between LSM and clinical parameters.

	r	*P*
Age	0.415	< 0.001
Sex	− 0.058	0.475
Height	0.409	< 0.001
Height SDS	0.224	0.005
Weight	0.521	< 0.001
Weight SDS	0.458	< 0.001
BMI	0.529	< 0.001
BMI SDS	0.481	< 0.001
Glucose	0.351	< 0.001
AST	0.364	< 0.001
ALT	0.291	< 0.001
TC	− 0.158	0.053
LDL-C	− 0.075	0.383
HDL-C	− 0.334	< 0.001
Triglycerides	0.221	0.009
TyG index	0.313	< 0.001
US-FLI	0.422	< 0.001

*BMI* body mass index, *SDS* standard deviation scores, *AST* aspartate transaminase, *ALT* alanine aminotransferase, *TC* total cholesterol, *LDL-C* low-density lipoprotein cholesterol, *HDL-C* high-density lipoprotein cholesterol, *TyG index* triglyceride glucose index, *US-FLI* ultrasonographic fatty liver indicator, *LSM* liver stiffness measurement.

### Association of clinical variables with LSM analysed by using simple linear regression analysis

Table 3 presents the results of the simple linear regression analysis of the association between the clinical variables and LSM. LSM had a positive association with age (β = 0.415,* P* < 0.001), height (β = 0.409,* P* < 0.001), height SDS (β = 0.224,* P* = 0.005), weight (β = 0.521,* P* < 0.001), weight SDS (β = 0.458,* P* < 0.001), BMI (β = 0.529, *P* < 0.001), BMI SDS (β = 0.481, *P* < 0.001), glucose (β = 0.291,* P* < 0.001), AST (β = 0.351, *P* < 0.001), ALT (β = 0.364,* P* < 0.001), TG (β = 0.221,* P* = 0.009), TyG index (β = 0.313,* P* < 0.001), and US-FLI (β = 0.422, *P* < 0.001), whereas HDL-C (β =  − 0.334,* P* = 0.009) showed a negative association with LSM.

**Table 3:** Association of clinical variables with LSM analysed by using simple linear regression analysis.

	B	SE	Standardized coefficients	*P*
Age	0.144	0.025	0.415	< 0.001
Height	0.023	0.004	0.409	< 0.001
Height SDS	0.157	0.055	0.224	0.005
Weight	0.020	0.003	0.521	< 0.001
Weight SDS	0.291	0.046	0.458	< 0.001
BMI	0.085	0.011	0.529	< 0.001
BMI SDS	0.323	0.047	0.481	< 0.001
Glucose	0.011	0.003	0.291	< 0.001
AST	0.010	0.002	0.351	< 0.001
ALT	0.005	0.001	0.364	< 0.001
HDL-C	− 0.021	0.005	− 0.334	< 0.001
Triglycerides	0.003	0.001	0.221	0.009
TyG index	0.449	0.117	0.313	< 0.001
US-FLI	0.196	0.034	0.422	< 0.001

*SE* standard error, *BMI* body mass index, *SDS* standard deviation scores, *AST* aspartate transaminase, *HDL-C* high-density lipoprotein cholesterol, *TyG index* triglyceride glucose index, *US-FLI* ultrasonographic fatty liver indicator, *LSM* liver stiffness measurement.

### Comparison of detection abilities of LSM and TyG index according to severity of NAFLD using receiver operating characteristic curve analysis

**Figure 1:**
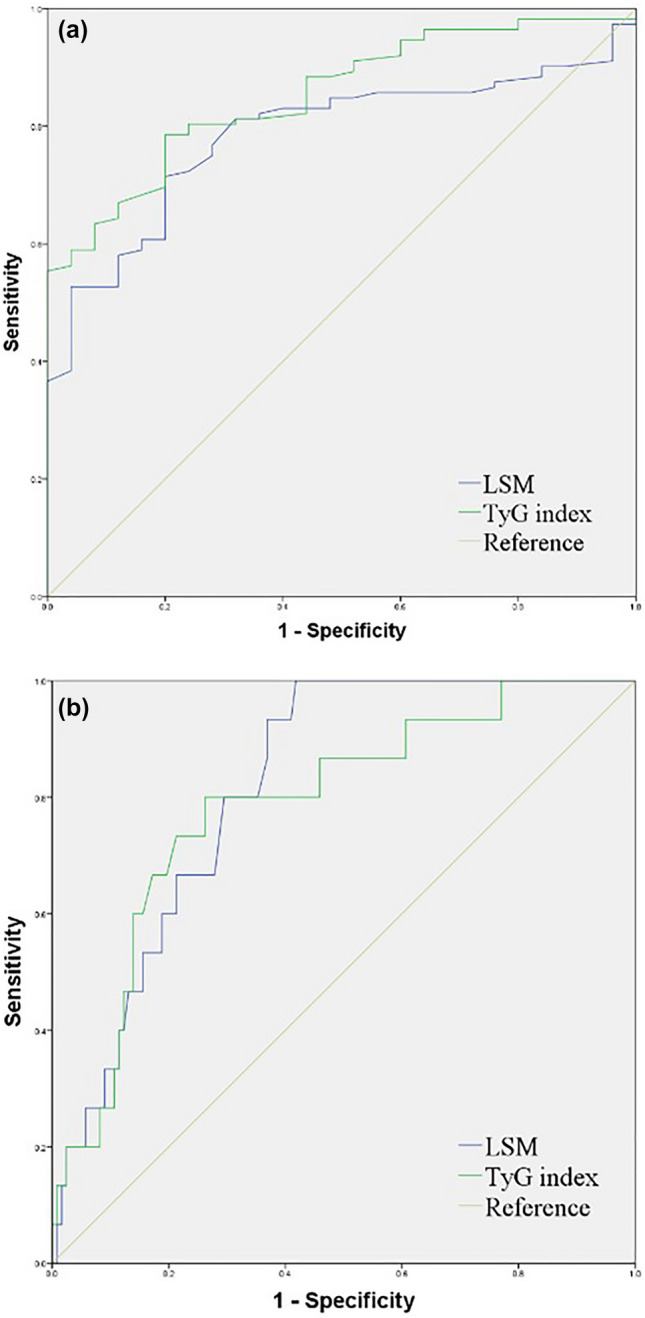
Comparison of abilities of LSM and TyG index to detect US-FLI ≥ 2 (**a**) and US-FLI ≥ 6 (**b**) using ROC analysis. *ALT* alanine transaminase, *TyG index* triglyceride glucose index, *TyG-ALT index* triglyceride-glucose-alanine aminotransferase index, *US-FLI* ultrasonographic fatty liver indicator, *ROC* receiver operating characteristic, *LSM* liver stiffness measurement.

Figure 1 and Table 4 show the results of the receiver operating characteristic (ROC) curve analysis of the abilities of transient elastography (TE) and TyG index to detect NAFLD (US-FLI ≥ 2) and high-severity NAFLD (US-FLI ≥ 6). For the detection of NAFLD, the area under the curve (AUC) of the TyG index and LSM was 0.852 (95% CI 0.785–0.920, *P* < 0.001) and 0.784 (95% CI 0.703–0.865, *P* < 0.001), respectively. Both the TyG index and LSM showed good abilities to detect NAFLD (a), and the TyG index was better than TE for NAFLD detection. The cut-off values for the detection of NAFLD were 8.265 (79% of sensitivity and 80% of specificity) and 3.905 (71% of sensitivity and 80% of specificity). When detecting US-FLI ≥ 6 (b), which is a more severe stage, the AUC of the TyG index and LSM was 0.786 (95% CI 0.668–0.905, *P* < 0.001) and 0.819 (95% CI 0.736–0.902, *P* < 0.001), respectively. Unlike the detection ability of NAFLD, LSM was superior to the TyG index in detecting increased NAFLD severity. The cut-off value for the detection of NAFLD was 8.810 (80% sensitivity and 74% specificity) for the TyG index and 4.165 (100% sensitivity and 58% specificity) for LSM.

**Table 4:** Comparison of detection abilities of 2D-SWE and TyG index according to severity of NAFLD using receiver operating characteristic curve analysis.

	US-FLI ≥ 2			US-FLI ≥ 6		
	AUC (95% CI)	Cut-off values (sensitivity/specificity, %)	*P*	AUC (95% CI)	Cut-off values (sensitivity/specificity, %)	*P*
TyG index	0.852 (0.785–0.920)	8.265 (79/80)	< 0.001	0.786 (0.668–0.905)	8.810 (80/74)	< 0.001
LSM	0.784 (0.703–0.865)	3.905 (71/80)	< 0.001	0.819 (0.736–0.902)	4.165 (100/58)	< 0.001

*NAFLD* non-alcoholic fatty liver disease, *NASH* non-alcoholic steatohepatitis, *TyG index* triglyceride glucose index, *TE* transient elastography, *ROC* receiver operating characteristic, *AUC* area under the curve, *CI* confidence interval, *LSM* liver stiffness measurement.

## Discussion

In this study, US-FLI was used to diagnose and evaluate NAFLD severity, and 2D-SWE was performed to measure liver stiffness. The TyG index was used to analyse the association between 2D-SWE and insulin resistance in NAFLD. The results showed that 2D-SWE was significantly associated with insulin resistance and had a good ability to detect and assess NAFLD severity.

Chronic liver damage caused by NAFLD results in liver fibrosis, leading to a stiffer-than-normal liver. Liver elastography is a promising diagnostic tool for objectively evaluating liver disease by reflecting pathological processes such as fibrosis^16^. Elastography has been used as a non-invasive evaluation tool for assessing liver stiffness for over 20 years^17^. Liver elastography can be divided into magnetic resonance elastography (MRE) and US-based elastography. MRE is an attractive non-invasive test that is more accurate for the detection and staging of liver fibrosis than US-based elastography methods but has the disadvantage of high cost^18^.

US-based elastography can be classified into TE, acoustic radiation force impulse (ARFI), and strain elastography. ARFI is divided into point shear wave elastography (p-SWE) and two-dimensional shear wave elastography (2D-SWE); 2D-SWE was used in this study^19^. US-based elastography has the advantage of being able to measure the overall stiffness or elasticity of the liver by evaluating the liver volume at least 100 times larger than that of a biopsy sample^20^. It is relatively inexpensive compared with MRE, making it a practical, non-invasive alternative for evaluating liver fibrosis in children in an outpatient clinic. TE is a one-dimensional elastography technique that was developed first, and its usefulness has been proven in many clinical studies^21^. In contrast, 2D-SWE is a relatively recently developed elasticity test method that provides two-dimensional elasticity images and has the advantage of being less affected by factors such as obesity and ascites than TE^22^. In 2D-SWE, liver stiffness is expressed as m/s, which represents the velocity of shear waves passing through the tissue or kPa based on Young’s modulus^23^.

According to studies on the standard value of normal liver stiffness by 2D-SWE in healthy children, Mărginean et al. suggested 3.72 ± 0.48 kPa as the average liver stiffness value, and Mjelle et al. suggested 3.3 kPa as the median liver stiffness^24,25^. The average liver stiffness value of 3.76 ± 0.28 kPa in healthy children presented in this study is in line with previous studies. It should be noted that the results of TE, p-SWE, and 2D-SWE differ when using the standard value of normal liver stiffness measured by US-based elastography. 2D-SWE showed significantly lower LSM values than TE and p-SWE^25^. In addition, among the studies using 2D-SWE, the standard value of normal liver stiffness differed depending on the US system and number of study participants^24^.

Studies on the usefulness of SWE for liver stiffness evaluation in the adult population are rapidly increasing; however, there are only a few studies on SWE in the paediatric population. Very few studies have compared liver elastography between children with NAFLD or NASH and healthy children using 2D-SWE. Mjelle et al. presented the cut-off values for liver stiffness that were obtained by 2D-SWE by age group (4.14 kPa for 3–5 years old, 4.59 kPa for 6–8 years old, 4.58 kPa for 9–11 years old, 4.88 kPa for 12–15 years old, and 4.66 kPa for 15–18 years old). There was no statistical significance between sex, and the cut-off values for boys and girls were 4.72 kPa and 4.63 kPa, respectively^25^. In the same context of diagnosing NAFLD with US alone, our study confirmed that paediatric patients with NAFLD can be discriminated from healthy children without a difference in the cut-off value of 2D-SWE suggested in previous studies.

Few studies have compared 2D-SWE results with histological findings from liver biopsies in children with NAFLD or NASH. Recently, studies suggesting cut-off values of 2D-SWE for liver fibrosis compared with histological findings obtained from liver biopsy have been continuously reported. According to the results of a study on the role of 2D-SWE in the differentiation of non-fibrotic liver disease and fibrotic liver disease in children with NAFLD using a recent liver biopsy, Hebelka et al. reported that 2D-SWE US was able to reliably distinguish between moderate/severe fibrosis and no/mild fibrosis in children with liver disease with excellent sensitivity and specificity^26^. For children with suspected or confirmed liver disease, the LSM for each stage of fibrosis is as follows: 5.0 kPa for F0, 5.0 kPa for F1, 5.8 kPa for F2, 7.5 kPa for F3, and 12.5 kPa for F4.

However, the results of 2D-SWE differ depending on the type of disease related to liver fibrosis. In the case of causes other than NAFLD, such as hepatitis, biliary atresia, and progressive familial intrahepatic cholestasis, the cut-off values according to the stage of fibrosis are as follows: 7.9 kPa for F0-1, 13.2 kPa for F2, and 21.7 kPa for F3^27^. Tran et al. also presented similar results, and the cut-off values of 2D-SWE to predict liver fibrosis resulting from causes other than NAFLD are as follows: 7.1 kPa for F0, 9.8 kPa for F2, 9.8 kPa for F3, and 13 kPa for F4^28^. Therefore, it is necessary to be careful when interpreting the results of US-based elastography because the cut-off value for diagnosing liver fibrosis differs depending on the disease.

In this study, non-invasive US-FLI, which can represent liver biopsy results, was used instead of liver biopsy to diagnose NAFLD and evaluate the severity of the disease. The results of our study showed that the association of LSM with age and BMI was similar to that reported in previous studies^29,30^. Since adolescents, insulin resistance, and obesity are risk factors for NAFLD, prepubertal non-obese children were set as a control group in this study to eliminate these confounding factors^31^. Therefore, age and auxological data, including height, weight, and BMI, were significantly different between the control and NAFLD groups. However, when only the NAFLD group was analysed, LSM still correlated with age and BMI. These clinical variables make it difficult to determine the LSM cut-off value for TE in paediatric NAFLD. Our study focused on analysing the clinical significance of changes in LSM values in children and adolescents with NAFLD rather than emphasising the presentation of cut-off values for LSM.

The strengths of this study are as follows: First, we studied the relationship with 2D-SWE for the first time, using US-FLI, a semi-quantitative measurement method. US-FLI is a useful test that accurately represents the severity of NAFLD-related liver biopsies^12,32^. According to the US-FLI score, which is a semi-quantitative evaluation technique, 2D-SWE was superior in the detection of the presence of NAFLD, which was more detectable with worsening severity. Until now, conventional US has been used as the first step in evaluating obese children and diagnosing NAFLD, and it is reasonable to perform elastography afterwards. Therefore, the significant association between US-FLI and 2D-SWE shown in our study is a necessary result suggesting the need for 2D-SWE examination at an appropriate time after screening for NAFLD by US-FLI in clinical practice. Second, this study suggests an association between 2D-SWE and insulin resistance. It is reasonable to infer that an increase in 2D-SWE in NAFLD is associated with an increase in insulin resistance. 2D-SWE had excellent detection ability for US-FLI ≥ 2 and ≥ 6. However, there were differences in clinical usefulness for each severity. In US-FLI ≥ 2, which is a criterion for diagnosing NAFLD, the TyG index was slightly more useful than LSM, but in US-FLI ≥ 6, where the severity worsened, LSM had better detection ability than the TyG index. In our study, LSM measured by 2D-SWE in children was correlated with the TyG index and suggested the ability to detect NAFLD as well as changes in severity. Our results provide the basis for LSM studies in the paediatric population. To our knowledge, this study is the first to show an association between 2D-SWE and insulin resistance using the TyG index.

The limitations of this study are as follows: First, liver biopsies were not performed to differentiate NAFLD from the different stages of liver fibrosis. The LSM values presented in previous studies cannot be compared with the participants in this study, and consequently the cut-off values presented in this study cannot be applied clinically. Although it has limitations compared with liver biopsy, US-FLI, which has been verified as a semi-quantitative evaluation tool to evaluate changes in LSM according to the severity of NAFLD, was sufficient to derive the study results. Second, as mentioned above, the control group and the study group had different ages and BMIs due to the characteristics of the research group. In the future, it will be necessary to design studies to classify age and BMI. Third, since the participants of this study were Korean children and adolescents, additional research is needed to apply the cut-off values and insulin resistance characteristics of 2D-SWE for NAFLD detection to the general paediatric population.

In conclusion, 2D-SWE not only showed good detection ability for NAFLD in children and adolescents but also showed better detection ability as NAFLD severity worsened. Importantly, 2D-SWE showed a significant association with the TyG index. Because insulin resistance is an important pathophysiological mechanism of NAFLD, 2D-SWE can be regarded as a reasonable tool for diagnosing NAFLD and monitoring disease progression in clinical practice. Further studies are needed to identify the role of 2D-SWE in the evaluation of liver steatosis and stiffness in the paediatric population.

## Methods

### Study population

This cross-sectional study included patients with NAFLD and healthy participants without NAFLD who successfully underwent 2D-SWE among patients under the age of 18 who visited a paediatric endocrine clinic for a health check-up from January 2021 to October 2022. This pediatric endocrine clinic treats endocrine diseases such as short stature, precocious puberty, and thyroid disease, as well as diseases associated with obesity such as diabetes, dyslipidaemia, and NAFLD.

Patients with NAFLD were diagnosed based on blood tests and ultrasound findings. ALT was used as a screening test for NAFLD, and levels ≥ 26 U/L for males and ≥ 22 U/L for females, which were suggested as threshold values for ALT for elevation in today’s youth study, were applied^33^. Patients with ALT levels above the threshold were tested for viral infection, including hepatitis A, B, and C, EBV, CMV, Wilson disease, and autoimmune hepatitis to determine the aetiology of liver disease. In addition, it was confirmed that there was no history of taking drugs related to hepatitis and no history of alcohol consumption. The US-FLI test was performed on all patients. NAFLD was finally diagnosed using US-FLI when all other diseases were ruled out.

Healthy controls were prepubertal children with normal ALT values and normal weight with a BMI between the 5th and 85th percentile. All of them had no abnormal metabolic diseases such as diabetes mellitus, dyslipidaemia, and hypertension, and there were no findings of renal disease or infection. And there was no history of alcohol consumption. Healthy controls underwent US-FLI and 2D-SWE for research purposes, with patient and guardian consent.

### Measurements of clinical and biochemical parameters

Auxological data including height, weight, and BMI, and biochemical data including serum glucose, AST, ALT, TC, HDL-C, and TG were examined within 1 week of the day US-FLI and TE were performed. Height was measured to the nearest 0.1 cm using a Harpenden stadiometer (Holtain Ltd., Crymych, Wales, UK), and weight was measured to the nearest 0.1 kg using a digital scale. BMI was calculated as weight divided by height squared (kg/m2). SDS values for height, weight, and BMI were calculated using the LMS (L, lambda for the Box–Cox power for skewness; M, mu for the median; S, sigma for the generalised coefficient of variation) method based on the 2017 Korean national standards: SDS = [measured value/M]^1/L^/LS^34^. Venous blood samples were obtained after the participants had fasted for at least 8 h. Biochemical tests, including serum glucose, AST, ALT, TC, TG, and HDL-C, were performed using an automatic analyser (Hitachi 7600, Hitachi, Tokyo, Japan). LDL-C level (mg/dL) was calculated using the Friedewald’s equation^35^. TyG index was calculated as ln (fasting TG [mg/dL] × fasting glucose [mg/dL]/2)^36^.

### Evaluation of liver steatosis and stiffness

US was performed by a single experienced paediatric radiologist who was unaware of the participants’ blood test results. All participants were tested after an overnight fast for at least 10 h. An ultrasound machine (Philips Epiq 7, Amsterdam, Netherlands) was used with a C5-1 MHz convex probe or a C5-1 (frequency range from 1 to 5 MHz).

Diagnosis of NAFLD and disease severity were evaluated by US-FLI. US-FLI is a semi-quantitative measurement method that uses the following criteria to grade scores 2 to 8: intensity of the liver/renal contrast, posterior attenuation of the US beam, blurring of blood vessels, difficult visualisation of the gallbladder wall, difficult visualisation of the diaphragm, and focal reserve area^12^. The presence of liver/renal contrast is used for the diagnosis of steatosis and is graded with a score of 2 for mild/moderate steatosis and a score of 3 for severe steatosis. As an additional criterion, the items of posterior attenuation of the US beam, obstruction of blood vessels, difficult visualisation of the gallbladder wall, difficult visualisation of the diaphragm, and focal reserve area were scored as 1 point for presence and 0 points for absence, respectively. NAFLD was diagnosed when the US-FLI score was at least ≥ 2. US-FLI has excellent discriminative power for different steatosis grades^12,32^. In adult, US-FLI was significantly correlated with pathologic criteria for NASH, and US-FLI < 4 had a high negative predictive value to rule out a diagnosis of severe NASH^12^. The optimal cut-off point for predicting hepatitis in children with NAFLD has been suggested as a US-FLI score ≥ 6^13^. Because participants in this study did not have liver biopsies, NASH cannot be diagnosed. And because the US-FLI was based on adults who underwent liver biopsies, it could not be directly applied to the participants in this study. In addition, the pediatric study on US-FLI presented above could not be applied to this study because the biopsy results were not directly compared with US-FLI. This study attempted to express the severity of NAFLD objectively by using the characteristic that US-FLI is useful for distinguishing the severity of NAFLD.

Liver stiffness was measured by 2D-SWE using an Epiq series (Philips Healthcare, Netherlands).

We performed 2D-SWE according to the manufacturer’s instructions. The patients fasted for > 8 h before the test. The test was performed with the patients in the supine or slightly lateral left position, with the arm raised above the head to widen the intercostal space. The paediatric radiologist placed the probe on the skin between the ribs from the right axillary anterior line to the axillary midline to the 7th–8th intercostal space, 1.5–2.0 cm below the capsule of the liver. Measurements were made 6–10 times, and the median value was used as a representative value. Liver stiffness was expressed in kPa based on Young’s modulus^19^.

### Statistical analysis

The Statistical Package for the Social Sciences version 26.0 (IBM Co., Armonk, NY, USA) was used to perform statistical analyses. To determine the statistical significance between the groups, the Student’s t-test or Mann–Whitney U test were used for continuous variables. Pearson’s correlation tests were performed to examine the correlation between LSM and clinical parameters. A simple linear regression analysis was performed to investigate the association between clinical variables. We verified the ability of 2D-SWE to detect the presence and severity of NAFLD using ROC curves and compared the ability of 2D-SWE to that of the TyG index to detect each stage of NAFLD severity (US FLI ≥ 2 or 6). Continuous variables are reported as mean ± standard deviation, and statistical significance was set at *P* < 0.05.

### Ethics statement

This study was conducted in accordance with the Declaration of Helsinki and was approved by the Institutional Review Board (IRB No. 2021-01-002) of the Hallym University Gangdong Sacred Heart Hospital. Signed informed consent was obtained from all participants and their parents. This study was conducted in accordance with the Declaration of Helsinki.

## Data Availability

The datasets used and/or analysed during the current study available from the corresponding author on reasonable request.
